# Aryan Views of Doctors

**Published:** 1884-12

**Authors:** 


**The Aryan Views of Doctors One Thousand Years Before Christ.**—The Aryans were a remarkable race. Their views of their doctors are commendable even now. Billings gives some of these views. Susruta advises medical students, "that they must utterly abstain from love and from hate, from anger and laziness, and from greed for gain. They must pay consideration to their external appearances, and have care that their clothing is appropriate and cleanly. They must be servants to the truth. They must show the same respect to the Brahmins, to their teachers in medicine, to their friends, and to all who turn to them for help. The doctor must wear his hair short; his nails must be clean and closely cut; he must never leave his house except with his cane or sunshade; and above all, he must avoid any undue intimacy with women. He must be handsome, well-built, amiable, earnest, but without self-conceit, friendly, and full of spirit; his speech must be soft, yet encouraging, as that of a friend; his heart must be pure and honorable; he must be a patron of cleverness and sagacity, and must love his patients better than relations, friends or parents. One may have a fear of a brother, a mother, or a friend, but never of his doctor." Besides the theoretical instruction by lectures and books, the student was trained and practically by visiting patients, witnessing operations, assisting in the same, and practising upon models. Before he could begin the practice of medicine, each student was required to obtain the consent of the ruler having charge of the practice of medicine.—*Medical Journal.*

The black plague derived its name from the dark and livid color of the spots and boils that broke out upon the patient's body. It descended along the Caucasus to the shores of the Mediterranean, spread over the South, and only entered Russia after the rest of Europe was affected. It followed the caravans which came from China across Central Asia until it reached the shores of the Black Sea; thence it was conveyed to Constantinople by ships, because this was then the commercial centre between Asia, Europe, and Africa. In the year 1347 it reached Sicily, Italy, and Marseilles. The next year it spread to France, Germany, and England. It did not reach Russia till 1351, or four years after it was at Constantinople. Thus, like the cholera, it followed a regular route, which differed according to the lines of trade and commerce at the different periods. The populace attributed the plague to water poisoned by the Jews, and persecuted them infamously: 1,200 Jews were burnt in Mayence London lost 100,000 in one month; Avignon and Florence, each 60,000; Marseilles, 56,000; Paris and Norwich, each 50,000; Strasburg and Erfurth, each 16,000; Basle, 14,000; and Venice, 10,000 in one month.

Some people are always finding fault with Nature for putting thorns on roses; I always thank her for having put roses on thorns.

